# Investigating the concept of participant burden in aging technology research

**DOI:** 10.1186/s12877-020-1441-3

**Published:** 2020-02-12

**Authors:** Katarzyna Kabacińska, Nicole Sharma, Jeffrey Kaye, Nora Mattek, Boris Kuzeljevic, Julie M. Robillard

**Affiliations:** 1 Division of Neurology, Department of Medicine, University of British Columbia, B402 Shaughnessy, 4480 Oak Street, Vancouver, BC V6H 3N1 Canada; 2 Oregon Health & Science University, Portland, OR USA; 3 Clinical Research Support Unit - BC Children’s Hospital Research Institute, Vancouver, BC Canada; 4 BC Children’s and Women’s Hospital, Vancouver, BC Canada

**Keywords:** Participation, Research burden, Technology, Research ethics

## Abstract

**Background:**

Research participation burden, despite being an integral concept in research ethics, is not well-conceptualized in the context of the use of technology in research. This knowledge gap is especially critical for the older adult population as new technology solutions are increasingly embedded in clinical trials for this demographic. Our objective was to investigate how older adults conceptualize participation burden in contact for research participation and research trials using technology.

**Methods:**

We developed and conducted an Internet-based survey consisting of 22 multiple choice and Likert-scale type questions investigating older adults’ preferred means and frequency of being contacted about research opportunities, their willingness to use specific kinds of technology and their concerns regarding technology use in clinical trials. We received a total of 273 completed surveys from eligible participants aged 50 or older.

**Results:**

Older adults preferred to be contacted about research opportunities monthly, over email. Survey participants were least willing to use monitoring devices and their biggest concern was the security of the storage of information gathered by technology. This concern was positively correlated with age. Participants indicated a preference to use technology daily, in short sessions, preferably in a way that can be incorporated into their daily routine.

**Conclusions:**

Results from this work provide insights for the design of effective recruitment campaigns as well as technology interventions in clinical trials through minimizing the burden of research participation.

## Background

The burden of research participation is a concept integral to the ethical principle of justice. Research ethics guidelines state that investigators should aim to reduce the burden of research participation by ensuring that the benefits of research outweigh the risks and that the benefits and burdens of scientific research are distributed equally in the population [1]. While this concept is promoted in research ethics education, including in key national efforts such as the National Institutes of Health training module on Human and Animal Subjects, its interpretation is likely to vary across different populations and circumstances and extend beyond direct risk to participants. Potential participants base their decision on the analysis of potential benefits and burdens associated with a given study, as well as their own motivations and context [2]. Thus, gaining a better understanding of what is perceived as burdensome is critical to informing more effective research designs and recruitment campaigns.

The need for a clearer definition of participation burden has been expressed by the research community. Ulrich, Wallen, Feister and Grady [3] point out that without empirical data and a better understanding of how participants conceptualize burden, we are unable to successfully address the problem of reducing perceived participation burden in the context of clinical research. Several efforts have been made to deepen the understanding of participation burden. Based on interviews with clinical trials’ participants, Ulrich and colleagues [2] identified categories describing the benefits and burdens of participation in research including physical, psychological, economic, familial and social dimensions. Building on this emerging conceptual framework, Lingler et al. [4] developed a tool for assessing perceived burden (Perceived Research Burden Assessment - PeRBA). They further divided the previously defined categories into pre- and post-enrollment in order to better capture the decision-making process of potential participants.

As health applications of new technologies are being developed, their use in clinical research increases. Nebeker and colleagues [5, 6] suggest that modern clinical research is facing a paradigm shift that provides new and fast ways of obtaining large quantities of accurate data, but also brings about potential threats, including a contribution to burden. As many scholars point out, the application of research methods including the use of tracking, mobile sensing devices and other technology should be conducted in a mindful way with consideration of the unique ethical challenges such as using online storage, privacy, or issues of autonomy when utilizing tracking devices [5, 7–9]. Several studies have explored older adult attitudes towards specific devices (e.g., GPS positioning devices [10], wearable step counters [11]) as well as towards specific ethical issues (e.g., privacy [8, 12]) in the context of every day use, where the technology is intended to benefit the user. Similarly, an extensive body of work has been conducted on technology acceptance measures for these devices, including in older adult populations [13–15], but to date little research examines willingness to use technologies and the perceived burden of that use specifically in the context of research. Donnelly and colleagues [11] used an exploratory approach to perform a study with the use of watches measuring physical activity in a nursing home. Based on subsequent interviews with the residents they identified dimensions of burden such as limited understanding of the research, emotional load, adherence and invasion of privacy due to the research procedure [11]. These early findings support the feed for further empirical research in this area.

The rapid increase in the use of technology for data collection, combined with lack of empirical data on participation burden, results in anxieties related to the use of technology in research [5, 7, 8]. At the intersection of these issues, Nebeker and Torous [16] point out a lack of safety protocols or best practices in research using new technologies which makes it difficult for Research Ethics Boards to make consistent decisions. Evidence-based recommendations about the burden of technology in research are urgently needed to guide institutional research ethics boards in decision-making when faced with novel investigative paradigms and tools. Further, these recommendations will be invaluable for researchers wanting to design aging studies that utilize technology in recruitment, assessment, monitoring or as an intervention itself.

In addressing these challenges, it is important to raise the research participant voice, as one of the purposes of ethical guidelines is the protection of participants.

The present study builds on the framework developed by Ulrich et al. [2, 3] and further advanced by Lingler et al. [4] to investigate the perceptions of older adults on the use of technology in research. Specifically, this research aims to answer the questions: How do older adults conceptualize burden in; 1) contact for research participation; and 2) technology use in clinical trials?

## Methods

### Study design

We developed a brief survey instrument based on gaps in knowledge identified by a literature review. Some of the questions were in part based on Lingler et al.’s [4] PeRBA, which identifies validated dimensions relevant to participant burden. The PeRBA instrument was not used in full as our research question pertained to burden in the context of research related to the use of technology more generally, rather than as it related to a specific study. A portion of the survey queried participants about their willingness to use new technologies. We selected technologies based on types of devices (home-based or mobile) currently being used in clinical trials as identified on the National Institutes of Health website clinical trial database (clinicaltrials.gov, date of search: December, 2017). Medical equipment devices such as fMRI (functional magnetic resonance imaging) or CAT (computed axial tomography) scanners were excluded. The resulting survey (excluding demographic questions) consisted of 20 items clustered around the following themes: 1) participant information; 2) research participation preferences and 3) concerns about the use of technology. The full survey can be found in Additional file 1.

### Setting

The survey was developed on Qualtrics (Qualtrics, Provo) and administered as an online form distributed via email in June 2018.

### Participants

The survey was administered through the Research via Internet Technology and Experience (RITE) Program. The Oregon Center for Aging and Technology (ORCATECH) RITE Program is a pool of participants who take part in health-related, Internet-based studies. RITE cohort participants were recruited using three strategies: 1) recruitment from patients who were registered online through the university’s health care electronic medical record (EPIC EMR) system, 2) e-mail contact of volunteers who indicated their interest to participate in research project with ORCATECH and 3) advertising of the research participation opportunity on the website. The program’s goal is to better understand people’s health needs, how Internet-based research can improve health care, and what kinds of health care information technologies participants would be willing to use [17]. Participants received an email with a link to the survey and were asked to complete it in their own time. After removing the outliers (*n* = 4), the average completion time was roughly 6 min (356 s.).

### Statistical methods

We performed descriptive statistics tests using SPSS software (Version 25.0). To determine whether there is a relationship between demographic characteristics of our sample and different concerns about the use of technology, we carried out Pearson correlations. Linear regression was used to determine whether there were effects of age, gender, education on responses to questions.

## Results

### Participants

A total of 572 potential participants were invited to fill out the survey, and 313 participants opened the link and started filling out the survey. Of those, 9 participants did not finish the questionnaire and their data was excluded from the analysis. Since our population of interest was older adults, participants younger than 50 years old (*n* = 28) and who did not disclose their age were excluded from the analysis (total excluded, *n* = 31). After exclusions, the total number of participants was 273.

### Descriptive data

The participants’ age ranged from 50 to 91 years old with a mean age of 69 (SD = 8.64). 158 participants (58%) were female. The majority of the participants were retired at the time of the study (72%). The most frequently reported highest level of education completed was a graduate degree (38%), followed by Bachelor degree (31%) and high school (14%). One in five participants did not disclose highest level of education completed (20%). Full demographic information for our sample can be found in the Table 1.

**Table 1:** Demographic information about the sample

#	Category	Subcategories	Frequencies^a^	Percentage
1	Age	Range: 50–91	Mean = 69	
2	State of residence	Oregon	234	86.6%
		Washington	28	10.4%
		California	4	1.5%
		Utah, New Mexico, NY or Alaska	4	1.5%
3	Gender	Female	158	57.9%
		Male	115	42.1%
4	Cohabitants^b^	Alone	61	19.7%
		With spouse or partner	194	62.6%
		With a parent	3	1.0%
		With adult children	23	7.4%
		With dependent children	18	5.8%
		With brother or sister	2	0.6%
		With friend	1	0.3%
		With roommate	3	1.0%
		Other	5	1.6%
5	Marital status	Married	180	66.7%
		Never married	17	6.3%
		Divorced	33	12.2%
		Widowed	26	9.6%
		Living as if married	8	3.0%
		Separated	1	0.4%
		Other	5	1.9%
6	Ethnicity	Hispanic	6	2.3%
		White	248	96.1%
		American, American Indian or Alaska Native	1	0.4%
		Asian	2	0.8%
		Other	1	0.4%
7	Retired	Yes	185	72.5%
		No	70	27.5%
8	Economic status	All needs met, could afford luxuries	192	75.3%
		All needs met, could not afford luxuries	60	23.5%
		One or more of the basic needs were not met	3	1.2%

^a^ Some questions were not answered by all participants

^b^ Participants could select more than one option

### Main results

#### Experience with technology

The majority of participants reported having used a computer for 5 or more years (99, 95% CI: 96.4–99.6%). When asked about computer use confidence on a scale from 1 (total lack of confidence) to 5 (extremely confident), the majority of participants responded in the 4–5 range (79, 95% CI: 73.4–83.6%). iPhones and Android devices were the most popular choice of communication devices, (88, 95% CI: 82.8–91.2%) followed by flip phones (10, 95% CI: 7.0–14.8%).

#### Contact for research participation

Nearly half (43%) of participants reported having previously taken part in a clinical trial. A vast majority of participants (94%) preferred to be contacted about research opportunities by email and 84% did not have a preference regarding the person who contacts them (physician or research assistants). The majority of participants (81%) indicated that they would be very interested in research participation if the research concerned a medical condition they or their loved one suffered from. Fewer older adults (64%) responded “very interested” regarding participation in research for the advancement of general knowledge. The most popular frequency of contact with research opportunities was monthly with 47% respondents choosing this option, followed by every few months (28%) and weekly (21%). Detailed answer rates for each question regarding contact for research participation are found in Table 2.

**Table 2:** Contact for research participation questions and response results

#	Question	Responses	Number	Percentage (95% CI)
1	Aside from participation in RITE, have you ever participated in a clinical study or trial?	Yes	117	43.2% (37.4–49.1%)
		No	154	56.8% (50.9–62.6%)
2	How would you prefer to be contacted about research participation opportunities?	Phone	3	1.1% (0.4–3.2%)
		Email	253	92.7% (88.9–95.2%)
		Mail	12	4.4% (2.5–7.5%)
		Social Media	0	0.0%
		Other	5	1.8% (0.8–4.2%)
3	By whom would you rather be contacted about research participation opportunities?	My physician	23	8.5% (5.7–12.4%)
		Research assistants	21	7.7% (5.1–11.5%)
		I don’t have a preference	228	83.8% (79.0–87.7%)
4	How interested would you be in research participation if the research concerned a condition you or a loved one suffers from?	Very interested	218	81.3% (76.2–85.6%)
		Moderately interested	46	17.2% (13.1–22.1%)
		Not really interested	2	0.7% (0.2–2.7%)
		Not interested at all	2	0.7% (0.2–2.7%)
5	How interested would you be in research participation if the research concerned advancing general knowledge?	Very interested	174	64.2% (58.3–69.7%)
		Moderately interested	92	33.9% (28.6–40.0%
		Not really interested	3	1.1% (0.4–3.2%)
		Not interested at all	2	0.7% (0.2–2.7%)
6	How often would you like to be contacted about opportunities for research participation?	Weekly	58	21.2% (16.7–26.5%)
		Monthly	129	47.3% (41.4–53.2%)
		Every few months	75	27.5% (22.3–33.1%)
		Yearly	9	3.3% (1.8–6.2%)
		Never	2	0.7% (0.1–2.9%)

#### Concerns related to technology

**Fig. 1:**
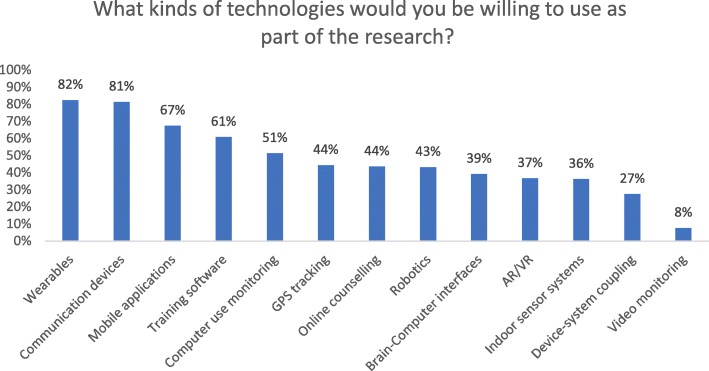
The willingness to use particular technologies while participating in research. Every participant could select any number of technology types

Respondents were asked which types of technology they would be willing to use in a clinical research trial context. The most frequently chosen types of technology were: wearables (e.g., step-monitoring, watch-like devices, 82, 95% CI: 77.5–86.5%), communication devices (e.g., mobile phones, 81, 95% CI: 76.3–85.5%) and mobile applications (e.g., health monitoring, cognitive training, 68, 95% CI: 61.6–72.7%). Technology chosen by the least participants (8, 95% CI: 5.1–11.5%) was video monitoring (e.g. in-home video monitoring). Response frequencies for each type of technology are found in Fig. 1.

**Fig. 2:**
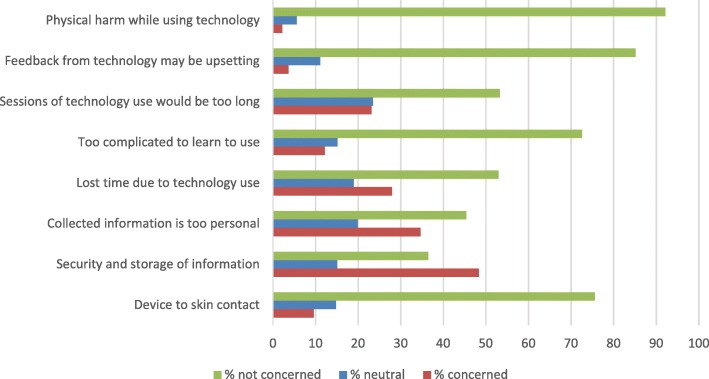
Answer rates for technology use concerns

Participants were presented with concerns about technology use in the form of sentences (e.g. “I’m concerned about the device contacting my skin”) and were asked to what extent they agree with the statements on a 7-point Likert scale (1 -“strongly agree”, 7-“strongly disagree”). The most concerning aspect of technology use was data security and information storage – 48% (95% CI: 41.5–53.4%) of respondents were concerned and 15% (95% CI: 11.4–20.0%) neutral. The second most concerning aspect was collecting information that is too personal, indicated by over a third of respondents (35, 95% CI: 29.0–40.3%). Less concern was reported about technology using up too much time – 28% (95% CI: 22.7–33.4%) were concerned, but over half of the participants (53, 95% CI: 47.2–59.1%) indicated lack of concern. Less than a quarter of participants (23, 95% CI: 18.6–28.6%) indicated that they are concerned about the length of technology use sessions, while 53% (95% CI: 47.2–59.0%) were not concerned. Most participants did not find it problematic to learn to use new technology (72, 95% CI: 66.8–77.5%) or having a device contact skin (75% responded in the 5–7 range, 95% CI: 70.5–80.6%). Similarly, most of the participants were not concerned about upsetting feedback from technology (85, 95% CI: 80.4–88.9%) or being physically harmed while using it (92, 95% CI: 88.9–95.1%). The detailed answer rates for questions about concerns can be found in Fig. 2.

The analysis revealed a significant effect of age (B = -0.27, Beta = − 0.229, *p* = 0.001) and gender (B = -5.85, Beta = − 0.142, *p* = 0.044) on security concerns. However, the model was very weak (adjusted R^2^: 0.045). There were no significant effects of age, gender and education on other types of technology concerns.

#### Technology schedules

When asked about preferred technology use schedules, nearly half (43, 95% CI: 36.9–48.7%) of respondents preferred using technology once daily and the most popular one-time session duration was 5–10 min (32, 95% CI: 26.1–37.1%). When given a choice between a lot of shorter use sessions, fewer longer sessions, a combination of both or continuous use throughout the trial, 48% (95% CI: 42.0–54.0%) of respondents indicated no preference. For those who did, the most popular answer was continuous use of a device throughout the trial that can be adapted to existing routine (19, 95% CI: 14.5–23.8%).

## Discussion

### Principal findings

Our goal was to capture the attitudes of a sample of older adults about participation burden and preferences related to the use of technology in clinical trials. We focused primarily on new technology that can be used at home or carried around such as wearables and virtual reality systems. Overall, we found that 1) our sample was generally accepting of the use of technologies such as wearables, communication devices and mobile applications in research with video monitoring being the least acceptable; and 2) the main concern about technology use in research expressed by our respondents was the security and storage of information. These findings contribute new knowledge about the preferences of the older adult population and can guide and inform future study designs and research recruitment efforts in a way that minimizes the participation burden and maximizes benefits.

#### Contact for research participation

Our results suggest that older adults prefer to be contacted by email and do not have a preference about who is contacting them. These two results are complimentary since email is a relatively impersonal mode of communication. These findings could be explained by the fact that our sample, the RITE cohort, is contacted primarily via email.

Recruiting older adults to participate in clinical research is challenging due to a number of factors including health concerns, mobility issues, reaching older populations and collaborating with institutions to reach those who do not live independently [18]. These barriers contribute to the underrepresentation of older adults in research [18, 19], which is problematic as people in this demographic have disproportionately more health needs [19]. We found that our participants were generally open to research participation and more willing to participate in clinical trials when they concern a condition that they/their loved ones suffer from than to advance general knowledge. Previous studies suggest that altruistic as well as personal reasons are a common motivation to take part in clinical research [20–22]. Almost half of the participants in our sample have previously participated in clinical trials which could influence their high interest and willingness to participate in future studies.

#### Technology use in clinical trials

We found that a large proportion of participants were willing to use a variety of different technologies in a clinical trial setting, with the exception of video-based monitoring. The unwillingness to use this technology in one’s house could be connected to concerns about data storage and security of personal or sensitive information. The use of tracking technology such as GPS is controversial due to ethical reasons [7, 8]. Landau and Werner [23] in their discussion of various aspects of the use of GPS tracking to increase the safety of older adults with dementia raise the question of whether safety should be prioritized over privacy and autonomy. The researchers also underline the importance of consulting individuals diagnosed with dementia to determine whether they are willing to use this type of technology [23]. Our findings contrast with a study by Nebeker and colleagues [6] aimed at measuring adults’ perceptions of mobile sensing devices after wearing them. The participants who used sensing devices in their study reported discomfort connected with the device touching their skin, such as irritation and interference with certain activities, rather than concerns about privacy [6]. This difference could stem from the age of participants, as we found a positive relationship between age and privacy concerns while using technology.

In terms of technology use schedules, participants favored continuous use of technology that is consistent with their established, daily schedules which suggests the least obtrusive technologies are preferred. This finding is consistent with the recent work of Donnelly et al. [11] which identified logistics and cognitive load (connected to charging and using wearable devices) as characteristics of burden in research using technology. As Hardy and colleagues [10] suggest, in order to make long-term participation in research using monitoring technologies possible, the device use effort must be minimized. Incorporating technology into the existing routine is one potential way to make participation more effortless and sustained, ultimately providing more ecologically valid data.

### Strengths and weaknesses of the study

This study is not without limitations. While our survey was based on already existing and validated frameworks, we cannot ensure its validity. Our sample was drawn from a participant pool that is technology-savvy enough to use the Internet and email on a regular basis and has agreed to be contacted for research participation. These factors may thus have influenced the results and skewed them in the direction of greater technology and research participation acceptance. Additionally, the response rate of this survey was lower than 50%. Although this response rate was expected based on other surveys distributed via the RITE cohort, self-selection to fill out the survey further limits the generalizability of the results. Another limitation is that the majority of participants identified as White and reported having a high socio-economic status and level of education. These variables have been previously linked to greater use of technology [24]. Future work in this area would benefit from querying a more diverse sample. We also note that the participants indicated their willingness of using the technology and participation in clinical research using the technology based largely on theoretical descriptions. We acknowledge that despite providing examples of particular technologies, conceptualizing what using a specific technology would entail could be difficult in some cases. However, when potential participants consider taking part in research, they often have to base their decision solely on a description during the consent process. Finally, it is difficult to reduce participant burden to narrow constructs such as the frequency of contact. Although we captured attitudes towards elements that have been previously identified to contribute to burden (Fig. 2), this study is intended to serve as a starting point to explore how these elements dynamically interact and contribute to the experience of burden as defined by participants.

Despite these limitations, results from the survey point to actionable recommendations for the use of technology in aging research. Beyond practical learnings from the data such as the preferences for monthly email communications and distributed technology usage schedules, we propose the following three recommendations: 1) when possible, researchers should incorporate technology solutions that are already familiar to older adults, such as wearables and communication devices; 2) where the introduction of an unfamiliar technology is necessary, research teams should consider a consultation process with potential participants to determine acceptable boundaries of technology use and address concerns prior to study launch, ideally prior to the finalizing of study design; and 3) when conducting studies that involve the collection of sensitive personal information, research teams should provide enhanced lay-friendly resources about data collection and analysis prior to or during the informed consent process, for example by showing visualizations of the types of data to be collected as well as clarity and transparency about proposed and eventual uses of the data.

## Conclusion

Our study provides a new perspective on older adults’ views of research participation. The fact that participants in our sample preferred receiving participation opportunities once a month can be used as a general guideline when contacting community-dwelling adults with research trial invitations. Our results suggest that older adults are willing to use various types of new technology in clinical trials, with the exception of video monitoring technology and that the biggest concern was the security of the information collected by the technology.

Beyond direct implications of each finding, results from the survey highlight first and foremost the need for future participant-centered, quantitative and qualitative work examining how participants experience technology-related burden, and how the most concerning risks identified in the present study can be mitigated. Further research is needed to investigate whether the anxieties about data provenance or storage influence older adults’ decision-making process about using technology in the context of privacy of use as well as use in clinical trials. We found that most of the participants in our sample were interested in clinical trial participation which could be a starting point for new research investigating whether the current strategies used to contact potential participants are adequate.

## Additional file

**Additional file 1.** Survey questions.

## Data Availability

The datasets used and/or analysed during the current study are available from the corresponding author on reasonable request.

## Abbreviations

CAT: Computed axial tomography
EMR: Electronic medical record
fMRI: Functional magnetic resonance imaging
PeRBA: Perceived Research Burden Assessment
RITE Program: Research via Internet Technology and Experience Program
