# Supplementary material for: Investigating the concept of participant burden in aging technology research
Source: BMC Geriatr. 2020 Feb 12;20:50. doi: 10.1186/s12877-020-1441-3 (PMC7017624; doi:10.1186/s12877-020-1441-3)
Supplement: Supplementary file 1 — Additional file 1. Survey questions. [file 12877_2020_1441_MOESM1_ESM.pdf]

Katarzyna Kabacińska, BA, Nicole Sharma, Jeffrey Kaye, MD, Nora Mattek, MPH, Boris Kuzeljevic, MA, Julie M. Robillard, PhD

Investigating the Concept of Burden in Technology Research

Please answer these survey questions about how YOU feel about participating in research. Your responses should not be limited to experiences with research you were previously involved with or are currently participating in. The goal of this research is to help us understand the preferences of research participants.

For the following questions, imagine a group is planning a new research study. They want your input on how best to recruit participants. *Note: You will NOT be recruited into any research studies based on your responses to this survey.*

Q1 Aside from participation in RITE, have you ever participated in a clinical study or trial?

- ○ Yes
- ○ No

Q2 How would you prefer to be contacted about research participation opportunities?

- ○ Phone
- ○ Email
- ○ Mail
- ○ Social Media
- ○ Other, please specify: ____

Q3 By whom would you rather be contacted about research participation opportunities?

- My physician
- Research assistants
- I don’t have a preference

Q4 How interested would you be in research participation if the research concerned a condition you or a loved one suffers from?

- Very interested
- Moderately interested
- Not really interested
- Not interested at all

Q5 How interested would you be in research participation if the research concerned advancing general knowledge?

- Very interested
- Moderately interested
- Not really interested
- Not interested at all

Q6 How often would you like to be contacted about opportunities for research participation?

- ○ Weekly
- ○ Monthly
- ○ Every few months
- ○ Yearly
- ○ Never

Q7 Imagine you are considering participation in clinical research. What kinds of technologies would you be willing to use as part of the research?

- ☐ Wearable devices (e.g. step-counting bracelet)
- ☐ Communication devices (e. g. smartphone)
- ☐ Video monitoring (e.g. cameras in your home)
- ☐ Indoor sensor systems (e.g. sensors in your house, automatic lights)
- ☐ Device – indoor system coupling (e.g. remotely controlled appliances)
- ☐ GPS tracking (watch with GPS)
- ☐ Mobile applications (e.g. health monitoring apps, cognitive training apps)
- ☐ Computer use monitoring (e.g. time on your computer, typing movements)
- ☐ Online counseling (e.g. Skype, teleconferencing)
- ☐ Brain-Computer Interfaces (e.g. a cap or headband that touches your head)
- ☐ Training software (e.g. computer games, video games)
- ☐ Augmented/Virtual reality (goggles with a small screen worn with a headband, used to play games, exercise)
- ☐ Robotics (e.g. interaction with social, pet-like robots)

Q8 Please rate to what extent you agree with the following statements when thinking about using technology in research or clinical trials:

Q9 I'm concerned about the device contacting my skin (e.g. while wearing step-monitoring, watch-like device)

- ◯ Strongly agree
- ◯
- ◯
- ◯
- ◯
- ◯
- ◯ Strongly disagree

Q10 I'm concerned about the security and storage of information that the technology is collecting about me

- ◯ Strongly agree
- ◯
- ◯
- ◯
- ◯
- ◯
- ◯ Strongly disagree

Q11 I’m concerned about the technology collecting information that is too personal (e.g. how much you weigh, your sexual activity).

- ○ Strongly agree
- ○
- ○
- ○
- ○
- ○
- ○ Strongly disagree

Q12 I’m concerned that the use of technology would take too much of my time (e.g. I will have to spend a lot of time using my computer/electronic devices more than I usually do).

- ○ Strongly agree
- ○
- ○
- ○
- ○
- ○
- ○ Strongly disagree

Q13 I’m concerned that the technology would be too complicated to use or learn how to use it.

- ◯ Strongly agree
- ◯
- ◯
- ◯
- ◯
- ◯
- ◯ Strongly disagree

Q14 I’m worried that sessions in which I would have to use technology would be too long (e.g. solving an exercise on a mobile application that takes a long time to complete).

- ◯ Strongly agree
- ◯
- ◯
- ◯
- ◯
- ◯
- ◯ Strongly disagree

Q15 I'm concerned that getting feedback from technology about my physical/mental health may upset me.

- ○ Strongly agree
- ○
- ○
- ○
- ○
- ○
- ○ Strongly disagree

Q16 I feel that I may be physically harmed while using the technology

- ○ Strongly agree
- ○
- ○
- ○
- ○
- ○
- ○ Strongly disagree

Q17 How often would you be willing to use a technology device during a clinical trial?

- ○ More often than twice daily
- ○ Twice daily
- ○ Once a day
- ○ Once every other day
- ○ Every two days
- ○ Once a week

Q18 Ideally, how long would you like a single technology-use session to last?

- ○ Less than 2 minutes
- ○ 2-5 minutes
- ○ 5-10 minutes
- ○ 10-15 minutes
- ○ 15-30 minutes
- ○ I only want to use technology that doesn't require me to interact with a device (e.g. sensors in my home)
- ○ I only want to use technology that doesn't require much interaction (e.g. a watch I wear for the entire trial without having to charge it every few days).

Q19 Which type of technology use schedule would you prefer?

- ○ A lot of shorter use sessions
- ○ Fewer sessions, but longer
- ○ Combination of shorter and longer sessions
- ○ Continuous throughout the trial but part of my existing daily routine
- ○ I don’t have a preference

Q20 Imagine that results from this study will be available in 18 months. Would you wish to receive information about the results of the study?

- ○ Yes
- ○ No

Q21 How would you want to receive the information about the results of the study?

- ○ Regular mail
- ○ Email
- ○ Social media
- ○ A phone call
